# Estradiol Alleviates Elevated Temperature-Induced Damage in Yak Oviductal Epithelial Cells by Maintaining Endoplasmic Reticulum Calcium Homeostasis

**DOI:** 10.3390/ani15091305

**Published:** 2025-04-30

**Authors:** Xiaolin Ye, Meng Wang, Shantong Qiu, Yangyang Pan, Yan Cui, Sijiu Yu

**Affiliations:** 1 College of Veterinary Medicine, Gansu Agricultural University, Lanzhou 730070, China; yexl@st.gsau.edu.cn (X.Y.); wangmeng@gsau.edu.cn (M.W.); qiushantong1998@163.com (S.Q.); panyangyang_2007@126.com (Y.P.); 2 Gansu Province Livestock Embryo Engineering Research Center, Department of Clinical Veterinary Medicine, Faculty of Veterinary Medicine, Gansu Agricultural University, Lanzhou 730070, China; 3 Laboratory of Animal Anatomy & Tissue Embryology, Department of Basic Veterinary Medicine, Faculty of Veterinary Medicine, Gansu Agricultural University, Lanzhou 730070, China

**Keywords:** estradiol, oviduct epithelial cells, temperature, yak

## Abstract

Background: The oviduct is an organ that participates in multiple critical reproductive processes and provides essential nutritional support while maintaining a specialized microenvironment. It is particularly vulnerable to damage following heat stress-induced hyperthermia. Therefore, mitigating heat-induced damage to oviduct epithelial cells while preserving their physiological integrity under hyperthermia represents a critical therapeutic goal. Objective: This study aims to simulate the cellular damage state in yak oviduct epithelial cells (YOECs) under thermal challenge by increasing the incubation temperature of cultured cells, while observing changes in cellular injury upon supplementation with 17β-estradiol (E_2_), in order to explore the underlying cellular regulatory mechanisms involved. Results: After 48 h of exposure to 41 °C, YOECs exhibited elevated HSP70 and HSP90 protein expression levels, reduced OVGP1 protein expression, and increased apoptotic cells. Compared to the 41 °C group, the E_2_ + 41 °C group displayed decreased HSP70 protein levels, increased OVGP1 protein expression, and reduced apoptotic cell numbers. Additionally, changes in endoplasmic reticulum calcium ion (ER-Ca^2+^) distribution and fluorescence intensity variations in ER-Ca^2+^ regulatory proteins SERCA and IP3R3 were analyzed in the 37 °C, 41 °C, and E_2_ + 41 °C groups. The ER-Ca^2+^ distribution pattern in the E_2_ + 41 °C group remained similar to that of the 37 °C group. However, the fluorescence intensity levels of SERCA and IP3R3 proteins in the E_2_ + 41 °C group did not recover to levels comparable to the 37 °C group. Conclusion: These findings suggest that E_2_ may mitigate thermal challenge-induced cellular damage in YOECs by maintaining ER-Ca^2+^ homeostasis, thereby preserving cellular functionality under elevated temperatures.

## Simple Summary

The oviduct is an organ involved in multiple reproductive processes and responsible for providing nutrients and a microenvironment. Under hyperthermic conditions, the composition and levels of secretory proteins in oviduct epithelial cells undergo alterations, accompanied by a significant increase in the number of apoptotic cells. A series of changes can lead to phenomena such as abnormal early embryonic development and spontaneous abortion. Therefore, identifying the mechanisms that alleviate damage to oviduct epithelial cells under elevated temperature provides a foundation for maintaining normal reproductive processes. Intriguingly, E_2_ modulates cellular defense mechanisms by sustaining endoplasmic reticulum Ca^2+^ homeostasis. This discovery establishes a theoretical foundation for understanding strategies to address hyperthermia-induced reproductive impairments.

## 1. Introduction

Heat stress is one of the main factors affecting animal reproduction. When animals exposed to heat load are unable to effectively regulate their body temperature, they enter a state of heat stress, leading to unfavorable physiological changes [1]. Heat stress causes many issues in reproductive function, such as decreased oocyte quality and developmental abnormalities in early embryos [2,3]. Therefore, animals have great difficulty naturally regulating the effects of heat stress on reproductive function.

The oviduct is a critical reproductive organ involved in multiple reproductive processes. Oviductal epithelial cells (OECs) provide nutritional support and a specialized microenvironment for key reproductive events, including protein secretion, ovum/embryo transport, and early embryonic development [4]. Under thermal elevation, OECs undergo detrimental changes such as increased heat shock protein 70 (HSP70) and heat shock protein 90 (HSP90) protein levels, decreased oviductal glycoprotein 1 (OVGP1) protein expression and increased apoptosis cells [5,6]. These functional disruptions impair oviductal physiology, leading to reproductive disorders such as estrus irregularities and reduced fertilization rates [2,7]. Therefore, it is essential to investigate the mechanisms by which heat stress causes functional damage to OECs and to identify methods for alleviating this damage. HSP70 serve as hallmark proteins induced by elevated body temperature in oviduct epithelial cells, where elevated expression levels indicate cellular heat stress. OVGP1, a constitutive protein in oviduct epithelial cells, participates in critical reproductive processes such as fertilization, early embryonic development, and transient sperm storage. Reduced OVGP1 expression disrupts these functions, leading to reproductive impairments such as diminished fertilization rates [2,7]. Thus, elucidating the mechanisms underlying elevated-body-temperature-induced OEC dysfunction and identifying strategies to mitigate such damage represent essential directions for reproductive research.

Cellular organelles are differentially affected during hyperthermia. The endoplasmic reticulum (ER), responsible for synthesizing secretory proteins, experiences functional impairment under thermal challenge, leading to aberrant synthesis and altered profiles of specific proteins [8,9]. Beyond protein production, the ER serves as a primary Ca^2+^ reservoir within the cell [10]. These dual roles of protein synthesis and Ca^2+^ storage are intimately interconnected; dysfunction in one invariably impacts the other. Ca^2+^ directly modulates ER-dependent protein synthesis, and drastic fluctuations in ER-Ca^2+^ concentrations induce the accumulation of misfolded/unfolded proteins. This ER-Ca^2+^ dyshomeostasis triggers ER stress, disrupts proteostasis, and may ultimately activate apoptotic pathways [11,12].

A critical hormone regulating oviductal epithelial cells (OECs), 17β-estradiol (E_2_), stimulates the synthesis of key secretory proteins (e.g., OVGP1), promotes cellular proliferation, mitigates apoptosis, and modulates Ca^2+^-associated protein functions [13,14,15,16]. Studies indicate that heat stress reduces systemic E_2_ levels in animals [17] while inducing drastic dysregulation of intracellular Ca^2+^ distribution [18]. Therefore, it is possible that supplementing with E_2_ could mitigate the adverse effects of hyperthermia in OECs.

In addition, the reproductive effects of heat stress are more pronounced in high-altitude animals than in low-altitude animals, which is related to the animals’ adaptation to the high-altitude environment. Therefore, the yak is a suitable model for studying heat stress. Yaks have evolved specialized adaptations to minimize heat dissipation and thrive in high-altitude hypoxic environments [19,20]. However, this evolutionary advantage entails a trade-off: yaks exhibit compromised thermoregulatory capacity under thermal challenge. Consequently, they are prone to heat stress during summer seasons or febrile conditions, which severely compromises reproductive performance [21]. The oviduct serves as an organ that provides nutrients and a microenvironment for reproductive processes while exerting buffering and protective functions during reproduction. Therefore, selecting yak oviduct epithelial cells (YOECs) as a hyperthermal model for studying thermal-induced reproductive damage represents an appropriate experimental approach. The effects of E_2_ on OECs under hyperthermic conditions and their underlying mechanisms remain incompletely understood. This study postulates that E_2_ may sustain the physiological functionality of OECs under thermal stress conditions, potentially through mechanisms involving Ca^2+^ signaling pathways. Addressing this hypothesis could yield novel insights into maintaining normal OEC function under elevated temperature conditions.

## 2. Methods

### 2.1. Cell Isolation and Culture

Intact reproductive tracts (comprising bilateral ovaries, oviducts, and uteri) were collected from three estrus-phase animals at Xining Slaughterhouse between September and November 2024. Estrous stage assessment was based on three criteria: (1) the presence of at least one active corpus luteum on either ovary, (2) the absence of embryos in the oviducts, (3) no implanted embryos in the uterine lumen [5]. Following collection, these were rinsed with 0.9% physiological saline solution supplemented with 4% Penicillin–Streptomycin (P4333, Sigma-Aldrich, Darmstadt, Germany) at 4 °C, then transported to the laboratory within insulated containers maintained at 4 °C with the same antibiotic-containing saline solution for subsequent processing [22].

YOEC collection and primary cell culture procedures were conducted as described previously. Detailed methodologies are provided in Supplementary Materials.

### 2.2. Western Blotting Analyses

The examined samples proteins expression was tested by Western blotting (WB). To assess cellular protein levels, cells were seeded in 35 mm dishes at a density of 2 × 10^4^/well for 24 h. Protein expression levels of oviductal glycoprotein 1 (OVGP1, NOVUS, Lone Tree, CO, USA, NBP1-76939), heat shock protein 70 (HSP70, Proteintech, Wuhan, China, 10995-1-AP), and heat shock protein 90 (HSP90, Proteintech, Wuhan, China, 13171-1-AP) were evaluated using antibody-specific protocols combined with standard Western blotting (WB) methodology. Technical specifications are elaborated in Supplementary Materials.

### 2.3. Immunofluorescence

Cells were seeded in 35 mm dishes of chambered slides at a density of 2 × 10^4^/well for 24 h. The cells were divided into three groups (*n* = 3), which were treated at 37 °C, 41 °C, and 41 °C + E_2_. To assess the purity of the cells and the endoplasmic reticulum Ca^2+^ import and pumping proteins sarcoplasmic/endoplasmic reticulum Ca(^2+^)ATPase (SERCA, CL488-67248, Proteintech, Wuhan, China), Type 3 inositol 1,4, 5-triphosphate receptor (IP3R3, Absin, Shanghai, China, abs151425), ryanodine receptor (RyR, Proteintech, Wuhan, China, 26968), 5-triphosphate receptor (IP3R3, Absin, Shanghai, China, abs151425), and ryanodine receptor (RyR, Proteintech, Wuhan, China, 26968-1-AP), we used localization and fluorescence intensity; for methods refer to the antibody instruction manual and see Supplementary Materials for detailed methods.

### 2.4. TUNEL Assay

To assess cellular apoptosis level, cells were seeded in 35 mm dishes of chambered slides at a density of 2 × 10^4^/well for 24 h. The cells were divided into seven groups, which were treated at 37 °C (12, 24, 48, 72 h), 39 °C (12, 24, 48, 72 h), 41 °C (12, 24, 48, 72 h), and TG + E_2_ (24, 48, 72 h). The samples were stained to detect apoptosis with reference to the instructions, as detailed in the Supplementary Materials.

### 2.5. Fluo-4AM Staining

To assess cellular Ca^2+^ distribution, cells were seeded in 35 mm dishes (353001, Corning, COR, NY, USA) of chambered slides (803460910, CITOTEST, Nanjing, China) at a density of 2 × 10^4^/well for 24 h. The cells were divided into three groups, which were treated at 37 °C, 41 °C, and 41 °C + E_2_ for 48 h. Intracellular calcium dynamics in heat-stressed YOECs (±E2 treatment) were analyzed using Fluo-4AM (Solarbio, Beijing, China, CA1190). A solution of 4 μM Fluo-4AM was covered with the samples and incubated in an incubator protected from light for 20 min. The samples were diluted in HBSS-buffered saline (Solarbio, Beijing, China, CA1190) and incubated again for 40 min. The samples were rinsed in HEPES-buffered saline (Solarbio, Beijing, China, CA1190). HEPES-buffered saline (Solarbio, Beijing, China, CA1190) was used for rinsing.

### 2.6. Endoplasmic Reticulum Tracker Staining

Samples were completely immersed in 400 nM ER tracker (Solarbio, Beijing, China, E2380) in an incubator and incubated for 20 min away from light before rinsing with HEPES. Images were collected using a live cell imaging fluorescence microscope (Delta Vision^TM^ Ultra, GE Healthcare Bio-Sciences Corp., Boston, MA, USA) and analyzed using Image J (v. 1.54f, NIH, MD, BU, USA).

### 2.7. Data Analysis

Image processing and grayscale quantification were performed using Image J software (v. 1.54f, NIH, MD, BU, USA) to extract grayscale values from both images and strip data, which were subsequently recorded and organized in Excel. Statistical analysis was conducted using SPSS (v. 25, MBI, Armonk, NY, USA) software. For datasets demonstrating normal distribution, parametric tests were implemented through SPSS. In comparative analyses between two groups, *t*-test selection criteria were applied. When Levene’s test for homogeneity of variance yielded *p* > 0.05, the pseudo-homogeneous variance was employed for significance determination. When Levene’s test resulted in *p* < 0.05, the Welch’s *t*-test (non-homogeneous variance) was applied for significance assessment. For comparisons between multiple groups, one-way analysis of variance (ANOVA) was utilized. Each experiment was independently repeated three times to ensure reproducibility. Statistical significance was set at *p* < 0.05. Results were visualized using GraphPad Prism 9 (GraphPad Software, Inc., San Diego, CA, USA).

## 3. Result

### 3.1. Identification of Yak Oviduct Epithelial Cells

YOECs were identified using the epithelial cell-specific expression protein cytokeratin 18 (CK 18). CK 18 antibody application to the YOECs resulted in a positive signal (Figure S1A).

### 3.2. Impact of Elevated Temperature on Yak Oviduct Epithelial Cells

**Figure 1:**
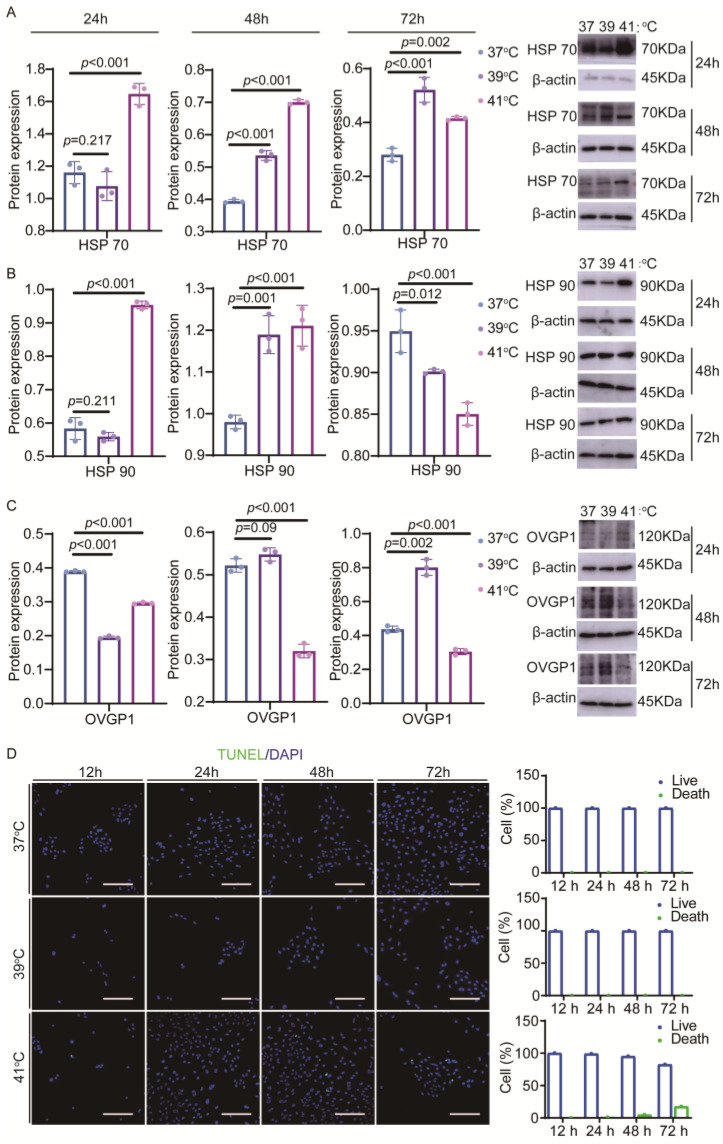
The establishment of a YOEC elevated-temperature model. (**A**–**C**) The Western blotting (WB) analysis of HSP90, HSP70, and OVGP1 protein expression levels. The blue series denotes the 37 °C group, the purple series corresponds to the 39 °C group, and the pink series represents the 41 °C group. Data are presented as mean values ± SEM (*n* = 3). Statistically significant differences: not significant *p* > 0.05, significant *p* < 0.05. (**D**) The analysis of apoptotic YOECs at various time points via TUNEL staining. The impact of 37 °C, 39 °C, and 41 °C groups on apoptosis in YOECs was assessed at various time points. Apoptotic cells were detected using TUNEL staining (green), and DAPI staining (blue) was employed to visualize the total nucleus in each sample. Fluorescence intensity quantification (scale bar = 100 μm) was normalized to viable cells (37 °C: 12 h apoptosis/live ratio 0%, 24 h apoptosis/live ratio 0%, 48 h apoptosis/live ratio 0%, 72 h apoptosis/live ratio 0%; 39 °C: 12 h apoptosis/live ratio 0%, 24 h apoptosis/live ratio 0%, 48 h apoptosis/live ratio 0%, 72 h apoptosis/live ratio 0%; 41 °C: 12 h apoptosis/live ratio 0%, 24 h apoptosis/live ratio 0.9%, 48 h apoptosis/live ratio 4.79%, 72 h apoptosis/live ratio 17.56%).

To investigate the effects of elevated temperature on YOECs, the present study evaluated protein expression patterns and apoptosis progression in YOECs under varying thermal conditions and temporal exposures. The experimental design incorporated multiple temperature gradients and time-course assessments to systematically analyze cellular responses to elevated temperature. The expression of hyperthermic stress signature proteins was evaluated in YOECs exposed to different temperatures (37 °C, 39 °C, and 41 °C) and varying durations (Figure 1). The expression of HSP70 protein was significantly increased in YOECs cultured at 39 °C (39 °C group) compared to YOECs cultured at 37 °C (37 °C group) at 48 h and 72 h (Figure 1A); the expression of HSP70 protein was significantly increased in YOECs cultured at 41 °C (41 °C group) at 24 h, 48 h, and 72 h (Figure 1A). HSP90 protein expression was significantly increased in the 39 °C group compared to the 37 °C group at 48 h (Figure 1B) as well as in the 41 °C group at 24 h and 48 h (Figure 1B). OVGP1 protein expression was significantly lower in the 39 °C group compared to the 37 °C group at 24 h (Figure 1C), and OVGP1 protein expression was significantly lower in the 41 °C group at 24 h, 48 h, and 72 h (Figure 1C). The effect of elevated temperature imbalance on apoptosis of YOECs was assessed using the TUNEL assay, which showed that the apoptosis rate gradually increased with time and temperature, and was 4.79% at 48 h and 17.56% at 72 h (Figure 1D). No apoptosis occurred in cells at other temperatures (Figure 1D). A comparison of the results of the above experiments showed that HSP70, HSP90, and OVGP1 protein expression, and the apoptosis of YOECs changed in the 41 °C group at 48 h. Therefore, these findings suggest that elevated temperature can simulate intracellular hyperthermal conditions, leading to abnormal expression of YOEC proteins and subsequent apoptosis.

### 3.3. Effect of Estradiol on Elevated Temperature Injury in Yak Oviduct Epithelial Cells

To investigate the role of E_2_ in mitigating elevated-temperature-induced damage in YOECs, the optimal concentration of E_2_ for enhancing OVGP1 protein levels was determined. WB analysis demonstrated that OVGP1 protein expression in YOECs treated with 1 nM E_2_ was significantly elevated compared to those treated with 10 nM E_2_ (Figure S2). The data demonstrate that the optimal E_2_ concentration for achieving peak OVGP1 levels is 1 nM.

**Figure 2:**
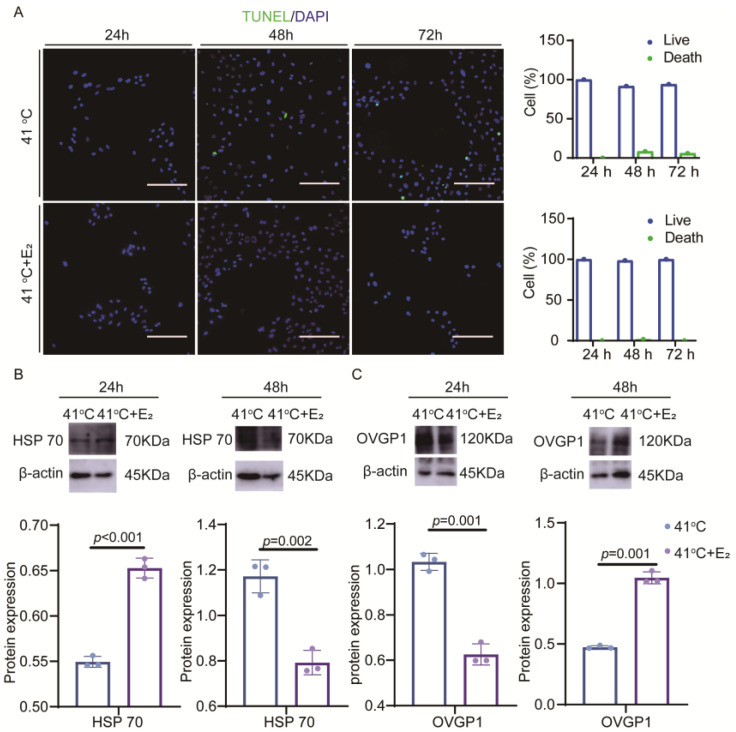
E_2_ alleviates the damage in elevated temperature YOECs. (**A**) The impact of 41 °C and 41 °C + E_2_ groups on apoptosis in YOECs was assessed at various time points. Apoptotic cells were detected using TUNEL staining (green), and DAPI staining (blue) was employed to visualize the total nucleus in each sample. Fluorescence intensity quantification (scale bar = 100 μm) was normalized to viable cells (41 °C: 24 h apoptosis/live ratio 0%, 48 h apoptosis/live ratio 8.3%, 72 h apoptosis/live ratio 5.9%; 41 °C + E_2_: 24 h apoptosis/live ratio 0%, 48 h apoptosis/live ratio 1.5%, 72 h apoptosis/live ratio 0%). (**B**,**C**) Western blotting (WB) analysis of E_2_ alleviates heat stress markers HSP70 and OVGP1 in YOEC protein expression levels. The blue series denotes the 41 °C group, the purple series corresponds to the 41 °C + E_2_ group. Data are presented as mean values ± SEM (*n* = 3). Statistically significant differences: no significant *p* > 0.05, significant *p* < 0.05.

YOECs cultured with 1 nM E_2_ supplementation (41 °C + E_2_ group) were compared with those maintained at 41 °C alone (41 °C group) to evaluate the impact of elevated temperature on cellular injury in YOECs. The results showed that apoptotic cells appeared; in the 41 °C group apoptosis rates measured 8.3% at 48 h and 5.9% at 72 h under control conditions. The 41 °C + E_2_ group demonstrated reduced apoptosis rates of 1.5% (48 h) and 0% (72 h), indicating significant amelioration of temperature-induced cellular damage (Figure 2A). It is clear that the conditions of the 41 °C + E_2_ group can alleviate the apoptosis caused by elevated temperature cultured.

**Figure 3:**
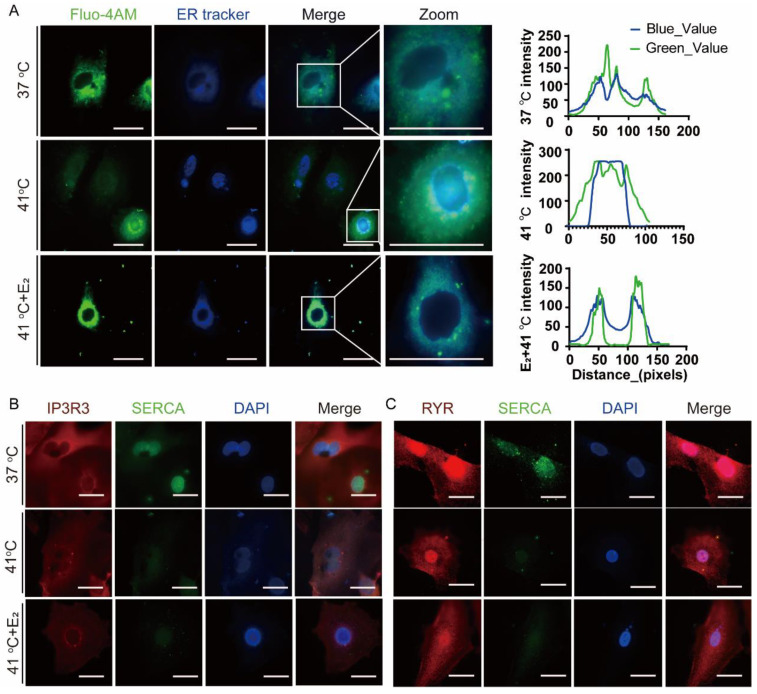
Estradiol alleviates elevated-temperature YOEC ER-Ca^2+^ distribution. (**A**) Intracellular Ca^2+^ levels were measured using the Fluo-4 AM probe under three conditions: 37 °C, 41 °C, and 41 °C + E_2_. Representative fluorescent images show co-localization of Fluo-4 AM (green) and ER marker (blue), with merged and zoomed views (scale bar = 25 μm). (**B**) The expression and subcellular localization of IP3R3 and SERCA in YOECs were examined through immunofluorescence analysis. The IP3R3 protein was visualized in red, SERCA protein was visualized in green, while the nucleus was stained in blue (scale bar = 25 μm). (**C**) The expression and subcellular localization of RYR and SERCA in YOECs were examined through immunofluorescence analysis. The RYR protein was visualized in red, SERCA protein was visualized in green, while the nucleus was stained in blue (scale bar = 25 μm).

To determine the effect of E_2_ on the expression of HSP70 protein in elevated-temperature YOECs, HSP70 protein expression was compared between the 41 °C group and the 41 °C + E_2_ group (Figure 2B). The HSP70 protein level was found to be significantly lower in the 41 °C + E_2_ group than in the 41 °C group at 48 h of incubation (Figure 3B). It can be seen that the 41 °C + E_2_ group significantly reduced the HSP70 protein expression of YOECs after 48 h of incubation.

To determine the effect of E_2_ on OVGP1 protein expression in elevated temperature YOECs, OVGP1 protein expression was compared between the 41 °C group and the 41 °C + E_2_ group (Figure 2C). It was found that the OVGP1 protein level in the 41 °C + E_2_ group was significantly higher than that in the 41 °C group at 48 h of incubation (Figure 2C). Combined with the above results, it can be seen that the addition of E_2_ incubation can effectively alleviate the damage of elevated temperature on YOECs.

### 3.4. Effects of Elevated Temperature on Endoplasmic Reticulum Ca^2+^ Distribution and Related Protein Expression of Yak Oviductal Epithelial Cells

To investigate the effect of E_2_ on Ca^2+^ distribution and related protein expression in elevated temperature YOECs, the Ca^2+^ signal distribution at 37 °C, 41 °C, and 41 °C + E_2_ were compared (Figure 3). It was found that Ca^2+^ in the 37 °C and 41 °C + E_2_ groups was mainly concentrated in the ER, and Ca^2+^ in the 41 °C group was less distributed in the ER and more distributed in the cytoplasm (Figure 3A). The comparison of the expression of major Ca^2+^-regulated proteins in the ER of the 37 °C, 41 °C, and 41 °C + E_2_ groups revealed that the expression of Ca^2+^-related proteins changed consistently in the 41 °C and 41 °C + E_2_ groups when compared with the 37 °C group, and the fluorescence intensities of SERCA and IP3R3 were reduced, and the fluorescence intensities of RYR were unchanged (Figure 3B,C).

## 4. Discussion

Understanding the mechanism by which E_2_ alleviates elevated-temperature-induced damage in YOECs provides a theoretical foundation for preserving reproductive health in thermally challenged animals. Ca^2+^, a ubiquitous intracellular signaling ion, critically governs cellular homeostasis, with its dysregulated distribution directly compromising cellular integrity and functionality [11,23]. This study demonstrates that E_2_ not only preserved ER-Ca^2+^ homeostasis in elevated-temperature YOECs but also effectively restored cellular viability.

### 4.1. Estradiol Can Alleviate Damage in Elevated Temperature Yak Oviduct Epithelial Cell

HSP70 is a hallmark biomarker of heat stress in animals, with its expression significantly upregulated under hyperthermia [17]. WB analysis in this study demonstrated dramatic induction of HSP70 in YOECs cultured at 41 °C, confirming elevated temperature activation. Intriguingly, HSP70 expression exhibited temperature-dependent kinetics, with earlier onset of upregulation at higher temperatures, consistent with the experimental paradigm. Previous studies report concomitant increases in HSP90 and reductions in OVGP1 levels in hyperthermic oviductal epithelia and luminal fluids [24], suggesting these markers as complementary indicators of thermal injury. The data corroborate these findings: YOECs at 41 °C showed significant HSP90 elevation and OVGP1 suppression, whereas 39 °C induced inconsistent expression fluctuations. Apoptosis serves as a key pathological metric of heat-induced cellular damage [25]. Notably, a significant escalation in apoptosis was observed when YOECs were exposed to 41 °C, with apoptotic cells demonstrating progressive intensification over time. The coordinated emergence of all hyperthermia biomarkers—HSP70/90 upregulation, OVGP1 suppression, and apoptotic activation—was consistently achieved following 48 h of sustained 41 °C exposure. This paradigm effectively recapitulates the pathognomonic heat stress profile that is characteristic of YOEC dysfunction under hyperthermia.

This study demonstrates that E_2_ effectively mitigates hyperthermia-induced cellular damage in YOECs. WB analysis demonstrated suppressed HSP70 protein expression in the 41 °C + E_2_ group compared to YOECs in the 37 °C group, indicating E_2_’s ability to antagonize HSP70 upregulation under elevated temperature. E_2_ enhances OVGP1 synthesis in YOECs, a pivotal protein essential for reproductive functions. Sustaining OVGP1 expression is therefore critical for maintaining normal reproductive activity [13]. The studying in the 41 °C + E_2_ group revealed elevated OVGP1 levels in YOECs, demonstrating that E_2_ not only promotes OVGP1 biosynthesis but also counteracts its elevated-temperature-induced suppression. The 41 °C + E_2_ group exhibited significantly lower apoptosis rates in YOECs compared to the 41 °C group, with levels comparable to those in the 37 °C group. This finding is consistent with the results observed in other studies where E_2_ was shown to alleviate apoptosis [26]. Combined with the restoration of multiple E_2_ modulated indicators in YOECs under elevated temperature, these findings indicate that E_2_ can mitigate the elevated temperature damage to YOECs.

### 4.2. E2 Restores Elevated-Temperature-Induced Endoplasmic Reticulum Ca^2+^ Dysregulation

This study further revealed that elevated temperature significantly depletes ER-Ca^2+^ levels in YOECs, whereas E_2_ supplementation effectively alleviate this decrease, restoring ER-Ca^2+^ content. Studies have shown that changes in ER-Ca^2+^ stores exhibit physiological fluctuations in response to systemic demands [27]. According to current research, ER-Ca^2+^ homeostasis is governed by three transporters: SERCA (sarco/endoplasmic reticulum Ca^2+^-ATPase), IP3R3 (inositol 1,4,5-trisphosphate receptor type 3), and RYR (ryanodine receptor) [28]. In the 41 °C group, YOECs exhibited not only ER-Ca^2+^ depletion but also dysregulated expression of these regulatory proteins: SERCA was downregulated, IP3R3 was upregulated, while RYR remained unaltered. Functionally, SERCA operates as the ER-Ca^2+^ ATPase pump driving ATP-dependent Ca^2+^ influx into the ER [29]. A reduction in SERCA protein expression diminishes the capacity to import Ca^2+^ into the ER, thereby decreasing its ER content. In contrast, IP3R3 and RYR mediate Ca^2+^ efflux, channeling ions from the ER to the cytosol or organelles [30,31]. Targeted analysis of these transporters provides critical molecular insights into how E_2_ preserves ER-Ca^2+^ homeostasis under elevated temperature. This experiment demonstrated reduced fluorescence intensity of SERCA and IP3R3 under elevated temperature in YOECs, while that of RYR remained unchanged. Intriguingly, the observed decrease in IP3R3 protein expression levels—contrary to its canonical stress response—may arise from rapid ER-Ca^2+^ depletion triggering a cytoprotective mechanism. Such adaptation could involve downregulating IP3R3 protein expression or modifying its structure to limit Ca^2+^ efflux, thereby preserving luminal Ca^2+^ reserves [32]. Collectively, these findings suggest that elevated temperature perturbs ER-Ca^2+^ homeostasis by modulating the expression of certain calcium related proteins.

This study demonstrates that E_2_ effectively mitigates elevated-temperature-induced depletion of ER-Ca^2+^ levels. E_2_ exhibits dual regulatory capabilities: modulating cellular Ca^2+^ dynamics and alleviating endoplasmic reticulum stress [16,33]. The ER serves not only as the primary intracellular Ca^2+^ reservoir but also maintains Ca^2+^ homeostasis critical for its protein-folding microenvironment and secretory pathway proteostasis. The findings reveal a pivotal mechanistic insight: E_2_ may orchestrate synergistic protection against heat stress-induced pathologies by restoring ER-Ca^2+^ homeostasis, thereby concurrently resolving dysregulated calcium signaling cascades and proteostatic derangements. Since ER-Ca^2+^ homeostasis is critically regulated by SERCA, IP3R3, and RYR, we hypothesized that E_2_-mediated restoration of ER-Ca^2+^ content in heat-stressed YOECs might involve the modulation of these proteins’ expression levels. Contrary to expectations, comparative analysis of the 41 °C and 41 °C + E_2_ treatment groups revealed no statistically significant differences in the expression of these ER-Ca^2+^ regulatory proteins—a paradoxical finding that suggests E_2_’s rescue effects operate through non-transcriptional mechanisms, potentially involving functional fine-tuning of existing protein pools (e.g., phosphorylation, oligomerization) or indirect modulation of auxiliary Ca^2+^ buffering systems. This phenomenon might be related to the functional forms of proteins. For example, increased acetylation of SERCA2a significantly reduces SERCA activity, while SUMOylation exerts the opposite effect. The second kinase domain of SPEG acts on SERCA2a, with SPEG directly phosphorylating the Thr484 site of SERCA2a to enhance its Ca^2+^ transport capacity. Tyrosine nitration of SERCA2a plays a crucial role in its activity regulation, thereby reducing SERCA2a’s ability to modulate Ca^2+^ homeostasis and impairing its enzymatic function [34]. E_2_ can directly or indirectly regulate ER-Ca^2+^ associated protein activities by altering post-translational modifications. For instance, estrogen inhibits cAMP levels to reduce RyR2 phosphorylation and its Ca^2+^ efflux capacity, while promoting the phosphorylation of phospholamban (PLB), a key regulator of SERCA2a activity [35]. It is therefore hypothesized that these abnormalities might result from E_2_-induced modifications that directly or indirectly alter the functionality of ER-Ca^2+^-related proteins [26]. In summary, E_2_ appears to increase ER-Ca^2+^ content in elevated-temperature YOECs, with the regulation of post-translational modification patterns in ER-Ca^2+^ associated proteins likely serving as the critical mechanism underlying these changes.

While the model accounts for inter-individual variability via biological replicates (*n* = 3 donors), donor-specific epigenetic states could modulate E_2_ responses. Future studies using isogenic iPSC-derived models may further isolate genetic vs. environmental contributions. The investigation into the cytoprotective mechanisms of E_2_ against elevated temperature in YOECs indicates that ER-Ca^2+^ equilibrium maintenance plays a pivotal role. However, the protein interaction networks mediating E_2_-enhanced ER-Ca^2+^ mobilization during heat stress remain to be fully elucidated. Furthermore, the precise signaling pathways through which E_2_ restores ER-Ca^2+^ homeostasis to mitigate thermal injury require comprehensive exploration. Future research directions will focus on characterizing these critical molecular mechanisms and their potential applications in elevated temperature related reproductive impairments. The practical application of E_2_ in vivo faces challenges due to difficulties in precisely controlling its concentration range [36,37]. As a pleiotropic hormone, E_2_ exerts extensive systemic effects on animal organisms, lacking target specificity, which often leads to unintended side effects [37,38]. These limitations restrict the direct use of E_2_ as a therapeutic agent for maintaining reproductive health under heat stress conditions. Comprehensive investigations into E_2_ analogs or targeted E_2_ delivery systems are required to elucidate their effects on YOEC viability under elevated-temperature conditions. This will establish foundational references for future research on thermotolerant reproductive health management.

## 5. Conclusions

This study reveals that the ER-Ca^2+^ homeostasis regulated by E_2_ constitutes a critical mechanism for maintaining the viability of elevated-temperature YOECs. These findings underscore the pivotal role of cellular ionic equilibrium in preserving the reproductive functionality of YOECs under elevated temperature. Furthermore, the discovery establishes a conceptual framework for developing interventions to mitigate hyperthermia-induced reproductive impairments in animals, providing strategic guidance for future studies on thermoresistant reproductive management.

## Data Availability

The data used to support the findings of this study are available from the corresponding authors upon request.

## Supplementary Materials

The following supporting information can be downloaded at: https://www.mdpi.com/article/10.3390/ani15091305/s1, Figure S1. Identification of YOECs epithelial cell markers; Figure S2. Western blotting (WB) analysis of the effect of different concentration E_2_ to YOECs OVGP1 protein expression levels.

## Abbreviations

BCA	bicinchoninic acid
Ctrl	control
CK 18	cytokeratin 18
DAPI	4’,6–diamidino–2–phenylindole
ER	endoplasmic reticulum
ELISA	enzyme–linked immunosorbent assay
E_2_	estradiol
FBS	fetal bovine serum
HSP70	heat shock protein 70
OECs	oviduct epithelial cells
OVGP1	oviductal glycoprotein 1
RYR	ryanodine receptor
SERCA	sarco/endoplasmic reticulum calcium ATPase
treat	treatment
IP3R3	type 3 inositol–1,4,5–trisphosphate receptor
YOECs	yak oviduct epithelial cells
