# Supplementary material for: Estradiol Alleviates Elevated Temperature-Induced Damage in Yak Oviductal Epithelial Cells by Maintaining Endoplasmic Reticulum Calcium Homeostasis
Source: Animals (Basel). 2025 Apr 30;15(9):1305. doi: 10.3390/ani15091305 (PMC12070912; doi:10.3390/ani15091305)
Supplement: Supplementary file 1 [file animals-15-01305-s001.zip › animals-3535634-supplementary.pdf]

Additional file 1

Estradiol Alleviates Elevated Temperature-Induced Damage in Yak Oviductal Epithelial Cells by Maintaining Endoplasmic Reticulum Calcium Homeostasis

Co–Author Details:

Xiaolin Ye [1,2], Meng Wang [1,2], Shantong Qiu [1,2], Yangyang Pan [1,2], Yan Cui [1,2,3,*] and Sijiu Yu [1,2,*]

1 College of Veterinary Medicine, Gansu Agricultural University, Lanzhou 730070, China

2 Gansu Province Livestock Embryo Engineering Research Center, Department of Clinical Veterinary Medicine, Faculty of Veterinary Medicine, Gansu Agricultural University, Lanzhou 730070, China;

3 Laboratory of Animal Anatomy & Tissue Embryology, Department of Basic Veterinary Medicine, Faculty of Veterinary Medicine, Gansu Agricultural University, Lanzhou 730070, China

* Correspondence: cuiyan@gsau.edu.cn (Y.C.); sijiuy@126.com (S.Y.)

Catalogues

Materials and methods……………………………………………………….……….3

References………………………………………………………..…..…..……..8

Figure S1……………………………………………………..…..……………....9

Figure S2.…………………………………………………………..…………..…9

Materials and methods

Yak oviduct epithelial cells isolation and culture

Intact reproductive tracts (comprising bilateral ovaries, oviducts, and uteri) were collected from three estrus-phase animals at Xining Slaughterhouse between September and November 2024. Estrous stage assessment was based on three criteria: (1) presence of at least one active corpus luteum on either ovary, (2) absence of embryos in the oviducts, and (3) no implanted embryos in the uterine lumen [1]. Following collection, these were rinsed with 0.9% physiological saline solution supplemented with 4% Penicillin–Streptomycin (P4333, Sigma Aldrich, Darmstadt, Germany) at 4°C, then transported to the laboratory within insulated containers maintained at 4°C with the same antibiotic containing saline solution for subsequent processing.

Following repeated rinsing with 0.9% physiological saline containing 4% Penicillin–Streptomycin (P/S; 15140148, Gibco, WLM, USA) at 37°C in the laboratory, uterine tissues, ovaries, oviducts lacking active corpora lutea, and excess peritubular adipose tissues were removed. Tissues were repeatedly washed with 0.9% saline supplemented with 4% P/S at 37°C until the effluent became clear. All oviducts were longitudinally incised to expose the luminal surface, sectioned into 1–2 mm fragments, and rinsed with PBS (G4202, AG Biotechnology, Hubei, China) containing 2% P/S. To compensate for variability in abattoir derived specimens, all oviductal fragments were pooled in conical tubes and enzymatically digested with 0.25% trypsin (25200072, Gibco, WLM, USA). Digestion proceeded for 50 min at 37°C in a temperature controlled orbital shaker (150 rpm). The reaction was terminated by adding phenol red free DMEM/F12 medium (L340KJ, Basal Media, Shanghai, China) supplemented with 10%

fetal bovine serum (FBS; SA211.02, Min Hai Biotechnology, Lanzhou, China). The cell suspension was filtered through a 100 μm nylon sieve (BD Falcon 352360, Corning, COR, NY, USA), followed by centrifugation at 2,500 rpm for 15 min. Pelleted cells were resuspended in phenol red free DMEM/F12 containing 2% P/S and 10% FBS, then cultured in 75 cm^2 flasks (430641, Corning, COR, NY, USA) under 5% CO_2 at 37°C for 4 days. After adherent cells exhibited morphological stability, purification was performed using 0.25% trypsin EDTA. Cryopreservation was conducted in freezing medium containing 1% DMSO (67-68-5, Solarbio, Beijing, China), 2% FBS, and 7% DMEM/F12 [2].

Western blotting analyses

The examined samples proteins expression was tested by Western blotting (WB). To assess cellular proteins level, cells were seeded in 35 mm dishes at density of 2×10^4/well for 24 h.

The proteins of samples have to be extracted with Radio Immunoprecipitation Assay buffer (R0010, Solarbio, Beijing, China). The extracted samples were lysed at 4 °C for 30 min The supernatant was then collected. The used the bicinchoninic acid (BCA) protein assay kit (PC0020, Solarbio, Beijing, China) quantified protein concentration. The protein lysates and protein loading buffer (P1015; Solarbio, Beijing, China) were mixing then denatured at 100 °C for 10 min. Then used wet transfer method separated protein and transferred from the gel onto a PVDF membrane. The membranes were blocked for 1 h with milk. After that incubated with primary antibodies overnight at 4 °C. The primary antibodies are from proteintech (Wuhan, China), dilution ratio 1:1000 include: anti–heat shock protein 70 (HSP 70, Proteintech, China Wuhan, 10995-1-AP), anti–heat shock protein 90 (HSP 90, Proteintech, China Wuhan, 13171-

1-AP) The other primary antibodies are anti–Oviductal glycoprotein 1 (1:1000, OVGP1, NOVUS, Lone Tree, CO, USA, NBP1-76939)), anti–β–actin (1:2000, TA7018, abmart, Shanghai, China), a The following day, washed membranes with TBST six times for 10 min. Then incubated with the secondary antibody. The secondary antibodys are from Absin Bioscience (Shanghai, China), dilution ratio 1:8000 include: goat anti–rabbit IgG–horseradish peroxidase (abs20040), and Goat anti–Mouse IgG–horseradish peroxidase (abs20001). After that wash membranes 5min with TBST for 12 times. Finally, the membranes were visualised by chemiluminescence. An ECL detection kit (P0018FS, Beyotime, Shanghai, China) and the Amersham Imager 600 system (GE Healthcare Life Sciences Corp., Boston, USA) and semi–quantified using the Image J (v. 1.54f, NIH, MD, BU, USA) was used.

Immunofluorescence

Cells were seeded in 35 mm dishes of chambered slides at a density of 2×10^4/well for 24 h. The cells were divided into three groups (n = 3), which were treated at 37℃, 41℃, and 41℃ +E_2. To assess the purity of the primary samples and investigate the expression and localization of cytokeratin–18 (CK-18), sarcoplasmic/ endoplasmic reticulum $Ca^{(2+)}$ATPase (SERCA), type 3 inositol 1,4,5-triphosphate receptor (IP3R3), ryanodine receptor, (RyR) in the samples, the following process were performed. Samples were fixed in 2% paraformaldehyde at 37 °C for 30 min. Permeabilization was achieved using 0.5% Triton X–100 (ST797, China Shanghai, Beyotime) for 15 min, followed by blocking with 5% BSA for 40 min. Anti–CK–18 (1:200, T55028, China Shanghai, abmart), anti-SERCA, (1:250, CL488-67248, China Wuhan, Proteintech), IP3R3, (1:250, abs151425, China Shanghai, absin), ryanodine receptor, anti-RyR,

(1:250, 26968-1-AP, China Wuhan, Proteintech). 4',6–diamidino–2–phenylindole (DAPI, C1002, China Shanghai, Beyotime) staining was performed for 5 min to label cell nucleus. The stained samples were sealed using an anti–fading mounting medium (C1002, China. Shanghai Beyotime). PBST was used as the buffer in this experiment. Images were captured using a live–cell–imaging fluorescence microscope.

TUNEL assay

To assess cellular apoptosis level, cells were seeded in 35 mm dishes of chambered slides at a density of 2 × 10^4/well for 24 h. The cells were divided into seven groups, which were treated at 37℃ (12, 24, 48, 72h), 39℃ (12, 24, 48, 72 h), 41℃ (12, 24, 48, 72h), TG+E_2 (24, 48, 72 h). Samples were subjected to three washes with PBS. Please refer to the appendix for pre–experiments based on heat stress modeling and conditions under which estradiol enhances cell activity. The cells were fixing with 4% paraformaldehyde (P0099, Beyotime, Shanghai, China) for 30 min and subsequently permeabilized using 0.5% Triton X–100 (ST797, Beyotime, Shanghai, China) for 30 min. Then thoroughly washed with PBS. Following another PBS wash, the samples were added with TUNEL reaction mixture (C1086, Beyotime, Shanghai, China) at 37 °C for 1 h in dark. After a final wash, stained samples with 4',6–diamidino–2–phenylindole (DAPI, C1002, Beyotime, Shanghai, China). Then observed under a live–cell–imaging microscope (Delta Vision™ Ultra, GE Healthcare Bio–Sciences Corp., Boston, USA) and semi–quantified using the Image J.

Statistical analysis

Image processing and grayscale quantification were performed using Image J software (v. 1.54f, NIH, MD, BU, USA) to extract grayscale values from both images and strip data, which were subsequently recorded and organized in Excel. Statistical analysis was conducted using SPSS (v. 25, MBI, Armonk, NY, USA) software. For datasets demonstrating normal distribution, parametric tests were implemented through SPSS. In comparative analyses between two groups: T-test selection criteria, when Levene's test for homogeneity of variance yielded $p > 0.05$, the pseudo-homogeneous variance was employed for significance determination. When Levene's test resulted in $p < 0.05$, the Welch's t-test (non-homogeneous variance) was applied for significance assessment. For comparisons multiple groups,while one-way analysis of variance (ANOVA) was utilized. Each experiment was independently repeated three times to ensure reproducibility. Statistical significance was set at $p < 0.05$. Results were visualised using GraphPad Prism 9 (GraphPad Software, Inc., San Diego, USA).

References

1. Menjivar NG, Gad A, Thompson RE, Meyers MA, Hollinshead FK, Tesfaye D. Bovine oviductal organoids: a multi-omics approach to capture the cellular and extracellular molecular response of the oviduct to heat stress. BMC Genomics. 2023 Oct 27;24(1):646. doi: 10.1186/s12864-023-09746-y. PMID: 37891479; PMCID: PMC10605953.

2. Wang J, Pan Y, Wang M, Xu R, Han X, Ma R, Zhao L, Zhang T, Wang Y, Zhao T, Ding T, Baloch AR, Wang L, Cui Y, Yu S. Follicular fluid exosomes regulate OVGP1 secretion in yak oviduct epithelial cells via autophagy in vitro. J Cell Physiol. 2023 May;238(5):1020-1035. doi: 10.1002/jcp.30988. Epub 2023 Apr 4. PMID: 37013674.

Figures and legends

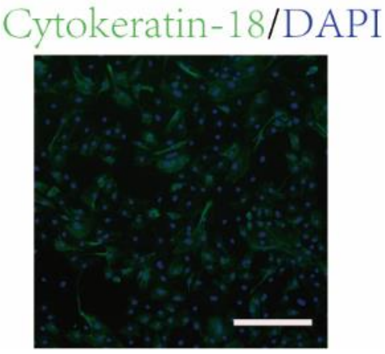

Figure S1 Identification of yak oviduct epithelial cell (YOEC). Immunofluorescence staining was performed to identification cellular. Cytokeratin-18 (CK-18) was visualized in green fluorescence, while nuclei were staining with DAPI (blue). (scale bar = 100 μm).

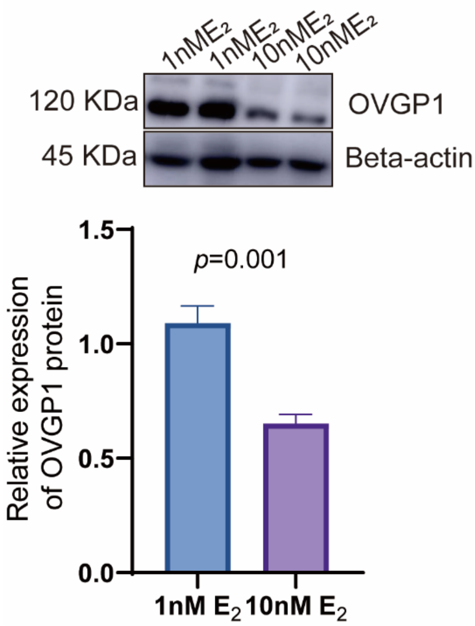

Figure S2 Western blotting (WB) analysis of the effect of different concentration E_2 to yak oviduct epithelial cells (YOECs). OVGP1 protein expression levels. Data are presented as mean values ± SEM (n = 3). Statistically significant differences (no significant $p > 0.05$, significant $p < 0.05$).
